# Developing and implementing a new health information technology innovation to improve patient safety in the Canadian context

**DOI:** 10.1177/08404704251346951

**Published:** 2025-06-05

**Authors:** Corinne M. Hohl, Arnold Ikedichi Okpani, Craig Kuziemsky

**Affiliations:** 1 12358 University of British Columbia, Vancouver, British Columbia, Canada.; 2 Vancouver Coastal Health Research Institute, Vancouver, British Columbia, Canada.; 3 3151 MacEwan University, Edmonton, Alberta, Canada.

## Abstract

Adverse Drug Events (ADEs) are unintended and harmful events related to medication use. Many ADEs recur because patients are unintentionally re-exposed to medications that previously caused harm. To help address this, we designed ActionADE, an interoperable Health Information Technology (HIT) that allows clinicians to communicate ADEs across health sectors. We completed ethnographic workplace observations and a systematic review to inform design. After piloting, we integrated ActionADE with the provincial medication dispensing database to alert pharmacists when patients seek to fill a prescription for the same or a same-class drug as one that previously caused harm. Co-design, application of clinically meaningful field labels and data standards, and integration with other health information systems were critical to ActionADE’s functionality and use. However, health system decision-makers need to proactively plan for how to spread and scale pilot project in the HIT ecosystem to ensure public benefit from successful innovation.

## Introduction

Medications have transformed the lives of Canadians suffering from medical and mental health conditions. With increasing medication use, Adverse Drug Events (ADEs) have unfortunately become common: each year, ADEs cause over 2 million emergency department visits and 700,000 hospital admissions in Canada.^1-5^ Ironically, medications intended to prevent and treat illness have become an important cause of emergency department visits, hospitalizations and deaths, and undermine advances in pharmaceutical care.^1,6^

Preventing ADEs is challenging as they occur as part of a sociotechnical system of systems involving clinical, organizational, technical, and other sub-systems. Widespread implementation of Electronic Medical Records (EMRs) has reduced some existing medication prescribing and administration errors, while also providing opportunities to address previously unrecognized safety issues.^6^ In 2019, we showed that 33% of patients presenting to acute care hospitals with ADEs suffered from *repeat* events due to re-exposures to the same or a similar medication as one that previously caused harm.^7,8^ While some re-exposures are medically appropriate (e.g., re-exposing an insulin-dependent person living with diabetes to insulin), 75% of re-exposures were deemed preventable.^9,10^

**Figure 1.**
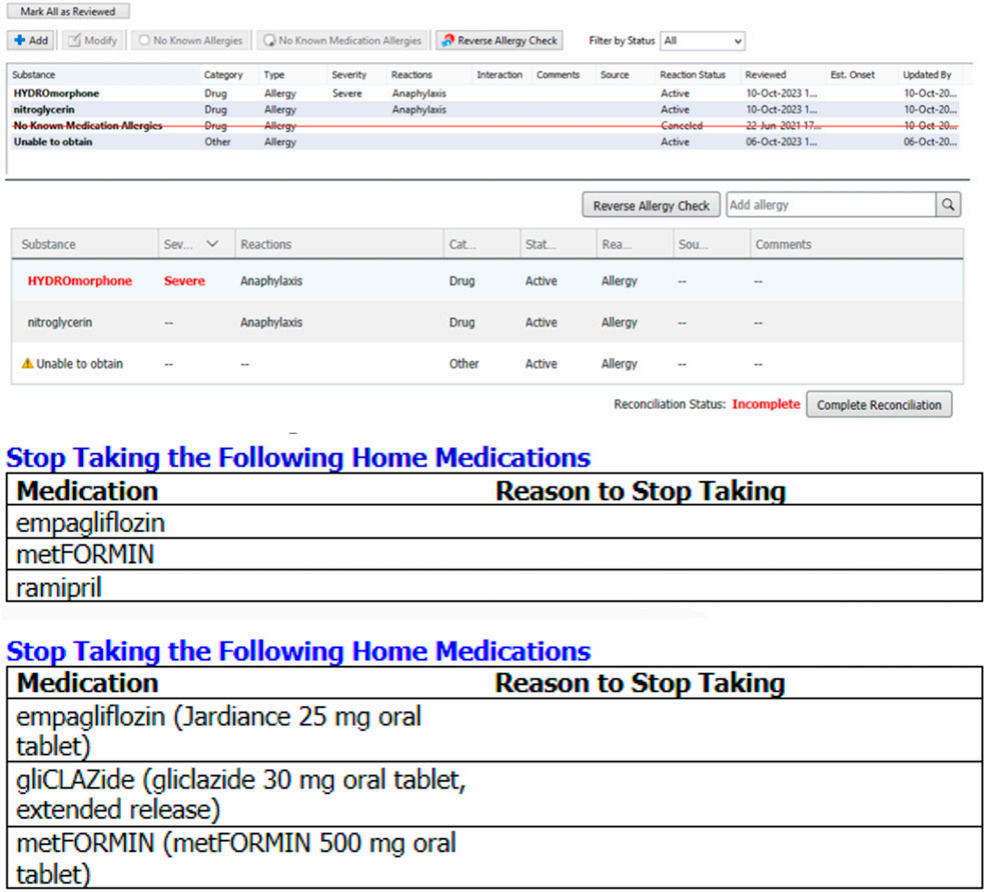
Screenshots of the allergy fields (top panel) and discharge summaries (bottom panel) of the EMR of the hospitals where GM had previously been admitted for metformin-induced lactic acidosis in 2020 and 2021 prior to his near-fatal third exposure to metformin in 2023.

Unintentional and preventable re-exposures occur because clinicians lack the means to effectively document ADEs within EMRs and then communicate them across siloed Electronic Health Record (EHR) systems and provincial drug dispensing databases used by community pharmacists.^11-13^ This means that ADE information does not follow the patient when transitioning from hospital to home, rehab, or long-term care (and vice-versa). Unintentional preventable re-exposure to culprit medications is harmful and can be life-threatening.^7,14-16^ Our prior research aligns with international studies to highlight the broad relevance of this newly described safety challenge.^14-17^ The vignette illustrates the real-life patient safety risks posed by non-interoperable health information systems. Figure 1 shows a screenshot of the allergy fields and discharge summaries from the patient’s previous admissions for the same adverse drug event.

“A male patient in his 50s [GM] with hypertension and diabetes was brought to our emergency department in severe pain. His electrocardiogram and chest X-ray were normal. The ED team was preparing to take him for a CT scan to rule out an aortic dissection when he suddenly stopped breathing. We intubated and began resuscitation. Shortly after, his bloodwork came back, revealing a blood pH of 6.67, the lowest we had ever seen in a living patient. Upon reviewing his medications, we discovered the cause: metformin-induced lactic acidosis. But what shocked us even more was learning that this wasn’t his first encounter with this condition. He had been admitted not just once, but two other times previously to different hospitals in our region with the same diagnosis. Several discharge summaries had clearly recommended discontinuing metformin. However, that information never made it to the patient’s community pharmacy which unknowingly re-dispensed the medication on multiple occasions. This nearly fatal re-exposure could have been prevented and he nearly died from this third recurrence.”

Anthony Lau, Emergency Pharmacist, October 2023

We took on the challenge of developing, implementing and scaling up a Health Information Technology (HIT) solution to address this ongoing problem. In this article, we summarize how we developed ActionADE, describe some of the challenges we encountered, and provide the key lessons learned for overcoming the last mile-problem in HIT implementation.

## Methods used to co-develop ActionADE to improve documentation and sharing of ADE information

ActionADE is a Canadian Institutes of Health Research (CIHR)-funded, evidence-informed HIT development, integration, and implementation project to prevent repeat ADEs by improving clinical documentation and sharing of ADE information across siloed health information systems.^18^

Recognizing that clinicians underreport ADEs,^19-22^ we started our work using ethnographic workplace observations to observe clinicians who diagnose, report, and treat ADEs using available health information systems. We completed over 350 hours of workplace observations. This allowed us to gain an understanding of workflow to inform the co-design of a user-friendly reporting interface.^23-25^ In parallel, members of our team completed a systematic review of 107 ADE reporting systems and EHRs to understand how existing health information technologies conceptualized and implemented ADE reporting.^26^ This foundational work informed focus groups and workshops with community and hospital pharmacists, family physicians, hospitalists, and emergency physicians to refine our understanding of reporting barriers.^27^ We asked clinicians to sort ADE reporting concepts and data fields to develop a minimum required dataset they would be willing to report, while providing sufficient information to enable decision-making.^23,24,27^ We called this the “minimum required dataset.” We then used a large case series of previously validated ADEs from prior prospective multi-centre studies^1-4^ to evaluate reporting standards and ensure we chose the most user-friendly terminology for our software.^28^ We then pilot-tested a paper-based form to inform design.^29^

**Figure 2.**
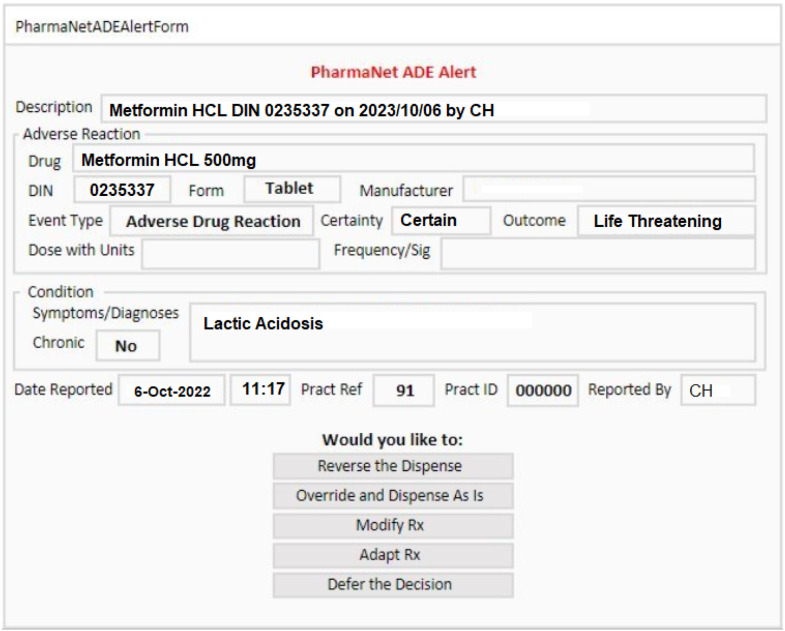
Example of an alert pop-up in one community pharmacy system using ADE information entered in ActionADE and shared with PharmaNet.

We co-designed ActionADE iteratively with end users, addressing identified barriers to ADE documentation and information sharing through design and integration.^27^ For example, integration with PharmaNet, the provincial drug dispensing database in British Columbia (BC), allowed ActionADE to pre-populate the patient’s medication dispensation history, such that a provider could click a dispensed medication to identify it as a culprit drug rather than having to take the time to enter the drug manually into the software. This and other design features, allowed us to optimize design and reduce the amount of time to report an ADE to a median of 119 seconds.^30^ PharmaNet integration also allowed us to send standardized ADE reports, tagged to the culprit drug identification number, from hospitals via PharmaNet to community pharmacies, where most culprit and same-class re-dispensations were occurring. We leveraged drug interaction software already implemented in PharmaNet, and co-developed conformance standards with the BC Ministry of Health for pharmacy software vendors to display the standardized ADE data in safety alerts when attempts to re-dispense the culprit or same-class drugs were made (Figure 2).^23,26,27,29,30^ We hypothesized that enhancing safety through systems integration, and by creating patient-specific medication-level alerts would increase clinicians’ motivation to report ADEs and minimize alert fatigue.^18^ This was confirmed in our discussion with healthcare providers who reported that the most important incentive for using an ADE reporting system would be to guide future care by creating safety alerts and enabling communication between providers. In addition to engagement with healthcare professionals, we conducted focus group discussions with groups of specific patient groups (e.g., seniors) to understand their expectations regarding needs and privacy concerns. These groups made clear that they expected relevant information that can impact patient safety—such as avoiding re-exposure to harmful medication—to be made available to all providers involved in the care of the patient.^31^

CIHR funding supported the evaluation of ActionADE’s impact on re-dispensations of culprit and same-class medications and subsequent health services utilization using a randomized controlled trial design.^32^ This provided us with a rare but important opportunity to evaluate the impact of sharing ADE information across health sectors using ActionADE on patient and system-level outcomes.^33^ This funding allowed us to implement ActionADE in nine acute care hospitals in Vancouver Coastal Health Authority, with a combined catchment population of 1.3 million. So far, preliminary evaluations of ActionADE have demonstrated a 270% increase in ADE reporting compared to the legacy reporting system, and a 33% avoidance of re-dispensations of culprit and same-class drugs in the intervention (information transmission) group.^30,34^ At the time of writing, the randomized controlled trial has recruited 70% of its planned sample size.

We provide three lessons learned for overcoming the last mile-problem in HIT implementation.

## Lesson 1: Clinicians need to intuitively understand data labels and fields without having to look up definitions

Clinicians who diagnose ADEs, and “hold” the relevant information and understand the pathophysiology of an ADE, may not share the same language as medical coders and health system technology developers. This impacts data field labelling and terminology.

Our systematic review of ADE reporting systems and EHRs revealed that many clinical information systems include “allergy” modules. However, allergies constitute only a small subset of clinically significant ADEs.^1,3,4,35^ Clinicians will not document non-allergy ADEs within allergy modules (Figure 1), as the data field label would be erroneous. Dedicated ADE fields are needed to enable clinicians document a broader range of clinically significant medication-related adverse events.

Many EHRs rely on non-standardized adverse reaction code sets provided by third-party vendors, with varying quantity, quality, and data standards preventing uniform documentation, extraction, and sharing of ADE data.^36^ If clinicians do not understand a code, they are likely to avoid documentation and underreport, or find a work-around to document in a free text field.^27^ Yet, free text data entry leads to the use of non-standardized abbreviations, ambiguous terminology, and miscommunication, and often precludes exchange of meaningful information or the design of useful safety alerts. Data standards matter.

After completing a systematic review of adverse drug event reporting systems which identified common data fields and standards prior,^26^ we asked clinical pharmacists to independently code 573 prospectively diagnosed ADEs using 4 commonly used international terminologies including ICD-10, SNOMED-CT, SNOMED-ADR, and MedDRA.^28^ We noted differences in coverage and usability.^28^ The ability of bedside clinicians to intuitively find and easily code an ADE was key to enabling reporting. End users needed to be able to document clinical syndromes (e.g., gastrointestinal bleeding), and update reports with diagnostic information (e.g., biopsy result) to confirm (e.g., non-steroidal anti-inflammatory induced gastric ulcer) or refute a medication-related cause when an alternative cause was subsequently made (e.g., biopsy demonstrating gastric cancer).^28^ We noted reporting hesitancy when diagnoses were uncertain, indicating the importance of being able to document and communicate certainty, and update reports with new information.^27^ Our work on data standards led us to adopt MedDRA terminology as the most comprehensive, accurate and user-friendly terminology with the least usability challenges, and design fields that could be updated by end users.^28^

During integration planning with our health authority’s EHR which uses SNOMED-CT, we needed to map MedDRA coded ADEs to SNOMED terminology. As SNOMED-CT is expansive, with over 350,000 concepts and 1.2 million synonyms, a more restricted code set was needed to facilitate searching and entry of relevant codes by clinicians. Our prior evaluation had demonstrated an unacceptably low coverage rate for SNOMED-ADR,^28^ so we used Snap2SNOMED to map ADEs that ActionADE had captured to date to SNOMED-CT to develop a smaller standardized SNOMED-CT-based value set.^37^ Our clinician reviewers were perplexed to encounter terms they had never heard of before (e.g., “solar erythema” instead of sunburn), and medical terms they were unfamiliar with despite years of medical and pharmacy practice (e.g., arginine vasopressin resistance instead of nephrogenic diabetes insipidus).

Ensuring that fields are appropriately labelled and that data standards are clinically usable, meaningful, and intuitively understood by frontline clinicians is critical prior to their adoption. As medicine and therapeutics evolve, standards will need to be updated, expanded and refined.^11^ HIT vendors and implementation teams should proactively seek out frontline clinicians who use systems to co-develop and validate data labels and fields, and update code maps to ensure usability.^11^ Semantics matter.

## Lesson 2: If you can add or leverage clinical functionality through integration, do it

Clinicians struggle with ever-increasing patient volumes, complexity and acuity.^12,27,35,38^ This means that patients wait longer to access care, and that clinicians spend less time per patient. Ensuring that health information technologies are adapted to clinical workflow and leverage integrations to facilitate work, not add to it, is paramount.

In 2019, the Canadian government implemented Vanessa’s Law which mandates the reporting of serious adverse drug reactions by hospitals to Health Canada to improve medication safety monitoring.^39^ Many hospitals implemented stand-alone patient safety learning systems to report serious adverse drug reactions to Health Canada, and embarked on extensive education campaigns to convince healthcare to use them even through prior work from other jurisdictions had indicated that underreporting would be extensive, and that legal reporting mandates and education campaigns would have limited success.^19-22^

ActionADE’s implementation and integration offered BC health authorities the opportunity to automate serious adverse drug reaction (a subset of ADEs) reporting to Health Canada, while improving cross-sectoral communication and patient safety.^18^ Our work with clinicians had indicated that allowing clinicians to report ADEs to safeguard patients would provide a strong clinical motivator to report ADEs compared to stand-alone reporting systems. Integration of ActionADE with Health Canada enabled automated data sharing of serious adverse drug reactions to fulfil institutional reporting requirements without burdening healthcare providers with duplicate data entry, enabling efficiency gains for clinicians without the need to build and deploy expensive new solutions. Despite this vision, the BC Ministry of Health adopted a stand-alone adverse event reporting system called the Patient Safety Learning System for serious adverse drug reaction reporting to Health Canada. After ActionADE’s integration with PharmaNet and Health Canada, evaluation of reporting volumes in both implemented systems demonstrated a 270% increase in reporting volume in ActionADE compared to the non-integrated Patient Safety Learning System.^30^ Hospitals that implemented ActionADE became national leaders in serious adverse drug reaction reporting to Health Canada.^40^ Simultaneously, community pharmacy responses to ActionADE alerts indicated that transmission of ADE reports from ActionADE to PharmaNet were associated with a 33% decline in re-dispensations of culprit medications.^30^ Creating safer systems motivates clinicians to report and enables high-quality data to be generated as a byproduct of clinical care.^18^ Integration matters.

## Lesson 3: Going through the research process represented a barrier to spread and scale of the HIT

HITs are routinely implemented at scale with little or no evidence of their effectiveness or safety, and with little clarity on their development process. In contrast, systems like ActionADE that are borne out of research projects, driven by frontline care providers and funded through competitive research awards, may face higher burdens of proof prior to scale up. Many never get past the pilot project, representing lost time, investment, and critically, missed opportunities to improve patient outcomes and experiences, population health, provider experiences, and system-level sustainability.^41^

ActionADE was conceived by researchers working across disciplines and universities, and in collaboration with a health authority, the Ministry of Health and professional organizations. It was co-funded by federal and provincial not-for-profit agencies to address a recently recognized patient safety issue.^18^ Countries that have been successful in advancing interoperability invested in the development of core components or “building blocks” such as ActionADE, that were common and standard across the health system.^11^ Despite promising preliminary data on the ActionADE building block demonstrated by improved ADE reporting rates and an important patient safety signal in a preliminary evaluation, ActionADE has yet to be transitioned to sustainability and scaled up provincially and across the country.^30,40^

This challenge to scale up is what is called the “Last-Mile Problem” where the full potential of clinical systems is not realized because of poor alignment with the sociotechnical environments where HIT are used.^42^ Overcoming the last mile-problem requires attention to be paid to the broader system of systems where HIT is used. As an example, IBM had advertised its Watson artificial intelligence platform as a revolutionary tool that would support clinicians in providing evidence-informed and personalized care for cancer patients. Despite high initial expectations and the promise of revolutionizing cancer care, IBM Watson struggled when implemented into real-life systems. While it could perform well on some tasks, it struggled to scale up to supporting the bigger system of tasks that define cancer care management.^43^

Digital health systems are part of a bigger system of systems where patients, providers, and organizational, technological, and policy systems interact and influence each other.^44^ Digital health implementation must account for system interactions and how they evolve over time. Spread and scale of HIT to solve system-level issues require deliberate planning, resource allocation, and iterative refinement to achieve their potential and enable public benefit. Building on success matters.

## Conclusion

Non-interoperable HIT can become an additional burden on already strained healthcare workers and can negatively impact patient care and safety. The ActionADE project offers several key lessons to support systems-based pursuits for overcoming the last mile-problem. First, we must meaningfully engage frontline clinicians and researchers in HIT design, integration and implementation to support usability and functionality of all end user needs. End user engagement is particularly important in planning system integration and evaluation of large-scale system purchases as they are designed without appropriate scientific evidence or input with respect to use and scale up in clinical settings. The failure of IBM Watson was case in point for what happens without a systems-based perspective on implementation. While success in pilot projects is common, governments need to ensure that processes and resources exist to refine, validate, and scale up safe and effective digital health solutions. HIT implementation is a journey, not a destination. Implementation and scale up are messy and not easy. HIT implementation is a learning system that will require constant review and rebuilding to retailor the technology to evolving health system needs.
